# Bioinspired Hybrid of Electrolysis and Fluidic Reaction (HEFR) Soft Actuator, Mimicking Natural Muscle Filament Motion With Electricity Generation Capability

**DOI:** 10.1155/abb/6656808

**Published:** 2026-07-06

**Authors:** Ramin Zakeri, Marjan Zahirinejad

**Affiliations:** ^1^ Mechanical Engineering Department, Shahrood University of Technology, Shahrood, Iran, shahroodut.ac.ir; ^2^ Chemical Engineering Department, Azad University of Karaj, Karaj, Iran, kiau.ac.ir

**Keywords:** actin, artificial muscle, chemical reactions, electrolysis, mechanism, myosin, soft actuators

## Abstract

This study employs the hybrid of electrolysis and fluidic reaction (HEFR) technique to investigate an experimental soft actuator model designed as an artificial myosin. Energy is generated through chemical reactions involving acetic acid (CH_3_COOH), bicarbonate (NaHCO_3_), and water (H_2_O), combined with chemical electrolysis, which powers the soft actuator to produce muscle‐like movements. Conversely, when the actuator relaxes under external stimuli, it can generate an electric current. The performance of the artificial myosin was assessed through three test cases. In the first case, the actuator, designed as a simple expanding mechanism, unfolded under pressurized fluid, demonstrating its ability to push weights ranging from 0 to 500 g in approximately 1 s. Additionally, in relaxation mode, the actuator produced electricity (~0.5 mW). The second test case examined the reciprocating motion of two opposing artificial myosins. Using the asymmetric principle of folding lines for each myosin and an edged actin on one side, this configuration achieved a 15 mm contraction per cycle within 2 s while also generating electricity during relaxation. In the third case, multiple artificial muscles were incorporated, combined with the asymmetric principle and variations in actuator stiffness, enabling contractile movements and curved rotations that mimic natural myosin function. This research demonstrates that integrating chemical reactions, electrolysis, and multiple soft actuators while applying the asymmetric principle can replicate myosin contraction similar to natural muscle while also enabling energy harvesting. The proposed approach holds significant potential for further development and optimization across various applications.

## 1. Introduction

Soft actuator methods have recently gained popularity as an alternative to hard actuators, particularly in complex environments, due to several advantages. One key benefit of soft actuators is their mechanical resilience and also flexibility, deformability, and ability to be miniaturized. Additionally, their compatibility with the environment and capacity to operate without causing harm to living beings or ecosystems make them highly desirable [1]. In certain approaches, soft actuators can even facilitate energy harvesting. Another notable advantage is their lower production cost, achieved through the use of simple materials and the capability to manufacture in dimensions as small as a few centimeters. Consequently, soft actuator methods are ideal for applications such as artificial muscles, micromanipulators, and microrobots [2].

Actuators derive their energy from various sources, including electricity, magnetism, photo‐ and thermally responsive actuation, chemical reactions, explosions, and pressure‐driven mechanisms. Electrical methods encompass technologies such as dielectric elastomers [3], piezoelectrics [4], and neurostimulation [5]. Magnetic methods use the Lorentz principle, which underpins the operation of many types of electric motors with various applications in different fields [6, 7]. Photo‐ and thermally responsive actuators include mechanisms driven by visible or near‐infrared light [8], as well as shape memory alloys (SMAs) [9], shape memory polymers (SMPs) [10], liquid crystal elastomers (LCEs) [11], and synthetic hydrogels [12]. Pressure‐driven approaches involve hydraulic and pneumatic systems [13, 14]. Finally, chemical reaction and explosion methods release energy through the interaction of two or more reactive materials [15, 16].

Each of these methods comes with its own set of advantages and disadvantages, making their suitability dependent on the specific application. Converting electrical energy into mechanical energy has notable benefits, including the potential to create flexible and stretchable strips, lightweight structures, and seamless integration with electronic circuits. Dielectric elastomer actuators, a well‐known method, rely on Coulombic attraction, where two flexible electrodes attract each other and compress the membrane. While the requirement for high voltage poses a significant drawback, the ability to harvest energy is a notable advantage. To enhance the efficiency and strain of this method, hydraulically amplified self‐healing electrostatic (HASEL) actuators have been developed. In these actuators, dielectric liquid within a shell is placed between flexible electrodes. When high voltage is applied to the electrodes, electrostatic Maxwell stress generates fluid pressure, leading to the shell’s contraction [17]. Mitchell et al. [18] introduced a simple method to create versatile and high‐performance HASEL actuators, ideal for untethered soft robots. Another approach in this category is the piezoelectric‐based actuator. This method generates voltage or electric charge when mechanical or vibrational forces are applied and produces small mechanical deformations under an electric field. Ma et al. [19] utilized piezoelectric actuation to enable the flapping‐wing motion of a small fly robot, capable of generating 1.3 mN of lift force. In summary, while energy harvesting from dielectric elastomers is feasible, the efficiency of this method remains significantly lower than that of magnetic approaches.

From another perspective, using electric motors based on the Lorentz force can generate mechanical work. This method has gained widespread adoption across various industries due to the technological maturity that enables its manufacturing in a wide range of sizes and torque levels. It also demonstrates satisfactory efficiency and capability for electricity generation in generator mode while likewise being applicable as an electric motor. However, its power‐to‐weight ratio remains relatively low compared to some alternative technologies. Calisti et al. [20] employed a DC servo motor connected to soft limbs to replicate octopus‐like movements. However, due to certain limitations of hard actuation methods like electric motors, magnetically responsive actuators have been introduced as soft alternatives. These actuators utilize sensitive materials—such as polymers, paper, gels, or fluids encased in shells—that can move under an external magnetic field. One significant advantage of this method is its rapid response time; speeds of up to 100 Hz have been reported, making it a promising option for applications like swimming robots, crawling robots, micropumps, and even energy harvesting [21, 22]. Another notable approach involves thermally responsive actuators, which include SMAs [23], SMPs [24], and LCEs [25]. While these methods, each with their respective pros and cons, can deform and return to their original shapes using thermal energy, they cannot generate electricity or thermal energy during reverse actuation. Wang et al. [26] demonstrated the SMA method in providing locomotion for an inchworm‐inspired robot. However, the rate of deformation and recovery is slower compared to methods like magnetically responsive actuation.

Additionally, sunlight‐activated actuators, based on carbon‐based materials or liquid crystal polymer networks, have their advantages. They can operate in environments without requiring internal energy sources, offering low energy consumption. For instance, Gelebart et al. [27] used this method to develop a light‐activated mill driven by high‐intensity light. This approach also shows potential for energy harvesting. Pneumatic and hydraulic methods are other well‐known techniques that transform energy into linear or rotational movements via fluids. These methods are particularly effective in large‐scale applications. However, challenges arise when miniaturizing actuators and their control systems. Su et al. [28] demonstrated that pneumatic actuators can achieve large bending angles and forces, while Galloway et al. [29] introduced hydraulically driven actuators for gripper robots with monolithic structures. None of these methods mimic nature, where the key mechanisms often involve chemical reactions and contraction processes [30–34]. Zakeri [30, 35, 36] introduced the hybrid of electrolysis and fluidic reaction (HEFR) technique, which combines fluid reactions (FRs) and electrolysis to release fluid pressure, converting it into mechanical energy, and some applications such as morphing of airfoil have been suggested. This hybrid approach closely resembles natural actuation. A comparison of the advantages and disadvantages of these methods is summarized in Table 1.

**Table 1:** Summarized points in related to pros and cons of different methods.

Method	Advantages	Disadvantages
Electrically responsive A. Dielectric elastomer [3]	Scalable, high power‐to‐weight ratio, so flexible, simple manufacturing	Low payload patience, need high voltage
Piezoelectric [4]	High power‐to‐weight ratio, simple	Small displacement
Magnetically responsive A [21, 22]	High harvest energy efficiency, high controllability, fast response time	High weight, complex to manufacturing in different scales, complex control system
Thermally responsive A. SMA [23]	Scalable, high power‐to‐weight ratio, large frequency response time	Low efficiency, high temperature ranges, complex manufacturing process
SPA [24]	Low cost, high elastic properties, different temperature ranges	Complex manufacturing process
Liquid metal [25]	Low voltage, low viscosity, high stretch ability	Complex control system, development is essential, low efficiency
Photo responsive A	Scalable, fast response time, simple control system, environmental friendly	Complex manufacturing process, low flexibility
Pressure driven A. Pneumatics [28]	High actuation force, light weight, no need return line	Not suitable for high load and control condition, complex control system
Hydraulic [29]	High actuation force, suitable for high load condition, stability, incompressibility	Need return line, complex control system, low efficiency in case of leakage
Chemical reaction of fluids/solids [30, 35, 36]	High energy releasing, high power‐to‐weight ratio, fast response time, high efficient method, harvest energy is possible, closer to natural actuation. Due to these advantages, this method is popular for researchers	Need control system, not easy to manifest, low life time, more research is essential

According to the studies conducted, while various methods have been developed—each with its own advantages and disadvantages—less attention has been devoted to studying and imitating nature. In this article, a combined method involving electrolysis and chemical reaction is employed to generate the energy required for contraction. Specifically, the energy source for movement is derived from the HEFR technique. By mimicking the behavior of myosin and actin, the fundamental contractile components of muscle fibers, an artificial model of muscle contraction has been created for the first time based on the asymmetric principle. This method also allows for energy harvesting when the artificial soft actuator is in motion.

## 2. Research Methodology of Current Artificial Muscle Technique

In this section, we examine the details of being’s movement, which is primarily generated by muscular systems that provide displacement and controlled motion. In this regard, biomimicry—drawing inspiration from natural mechanisms—offers significant advantages over conventional engineering approaches. Although the complete replication of natural systems is undoubtedly impossible, insights derived from this study can open a new perspective and shed light on innovative directions for our research. First of all, a series of studies were conducted to achieve a better understanding of the mechanism of muscle contraction, as reported in Section 2.1. Based on these studies, several key principles were identified. Subsequently, in order to evaluate the proposed mechanism, a number of experimental soft actuators were fabricated. The data obtained from these experiments were analyzed from both quantitative and qualitative perspectives, and the results are presented in Section 2.4. Some background information is necessary for Section 2.4, which is provided in Sections 2.2 and 2.3. From several perspectives, this research can be considered valuable. First of all, the research approach was conducted in parallel with the natural contractile mechanism, although with a lower level of detail. Despite this simplification, several advantages were achieved, such as significantly lower noise and weight compared to conventional rigid actuation systems, including electrical motors and other hard actuation mechanisms.

### 2.1. Natural Muscle Mechanism

**Figure 1:**
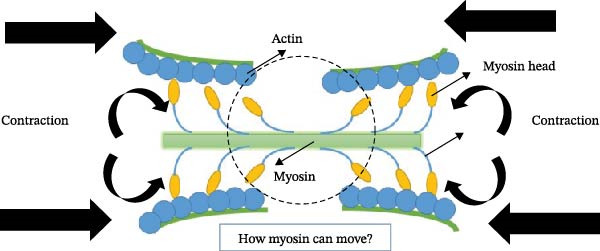
Natural filament mechanism considering the natural muscle contraction.

Movement without muscle contraction is undoubtedly impossible, and drawing on the existing natural mechanism is crucial for creating motion. By examining this mechanism, myosin’s role becomes evident as a fundamental component of an organism’s movement. However, the intricate workings of myosin remain poorly understood. Myosin moves in a curved path upward, binds to actin to form a cross‐bridge, pulls actin, and then moves downward to repeat the cycle. Overall, in organisms, the fluid movement will transfer energy, which is converted into work through diverse biological processes [31]. Muscles consist of numerous microfibers, each containing thousands of myofibrils. Each myofibril houses thousands of filaments, primarily made of two types of protein filaments: myosin and actin. These filaments play a pivotal role in muscle contraction. Surrounding each myofibril is intracellular fluid called sarcoplasm, which contains essential proteins, potassium, phosphate, magnesium, enzymes, and mitochondria. Mitochondria provide energy for contracting myofibrils via adenosine triphosphate (ATP) [32]. The core of contraction lies in myosin’s movement and its ability to attach to actin, forming a cross‐bridge, which is depicted in Figure 1. This connection facilitates the sliding motion of actin filaments, driving contraction. Calcium ions, sourced from the sarcoplasmic reticulum, stimulate myosin’s movement and enable contraction. Once contraction ends, relaxation occurs as calcium ions are absorbed and returned to the sarcoplasmic reticulum by calcium pumps, halting contraction. A critical question arises: How does myosin move flexibly, bind to actin (cross‐bridge), and transmit force? This area in biology has not been thoroughly studied, though several theories exist. Chemical reactions are undoubtedly key contributors to movement, and alterations in fluid concentration or pressure likely drive this process. This paper aims to mimic the natural muscle mechanism by employing the HEFR technique, which closely resembles natural muscle contraction. Criteria for the proposed method include energy harvesting and replicating the movement of myosin and actin as detailed below.

### 2.2. Introduction to HEFR Technique

In the HEFR mechanism, first proposed by Zakeri [30, 35, 36], the hybrid model combines the FR process with electrolysis, resulting in the production of significant discharge gases. These gases can be utilized as high‐pressure sources to generate mechanical movement. Electrolysis not only contributes to gas production but also increases the fluid’s temperature, thereby accelerating the chemical reaction rate. The FR process involves the combination of at least two fluids capable of producing gas when reacting together. For instance, fluid A, such as acetic acid (CH_3_COOH or vinegar), reacts with fluid B, such as sodium hydrogen bicarbonate dissolved in water (NaHCO_3_ + H_2_O, or a baking soda solution), to produce discharge gases like carbon dioxide (NaHCO_3_ + CH_3_COOH + H_2_O → CH_3_COOHNa + 2 H_2_O + CO_2_).

**Figure 2:**
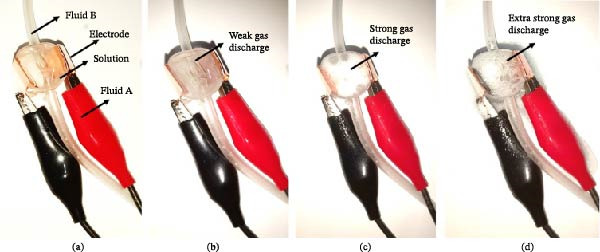
General HEFR technique mechanism and comparison of gas production in different modes. (a) Simple HEFR technique, (b) electrolysis of solution, (c) fluid reaction, and (d) hybrid reaction of electrolysis and fluid reaction.

In addition to the discharge gases produced through this chemical reaction, the electrolysis process generates additional gases. Electrolysis involves two metal electrodes and the application of an electric current within an electrolyte solution. To ensure sufficient conductivity, substances like sodium chloride (NaCl) are typically added to the solution. The electrolysis process results in the release of various gases, as seen in Figure 2, such as oxygen and hydrogen, as well as other byproducts (see Figure 2). Within the HEFR mechanism, the combined gases create pressurization, ultimately enabling the production of mechanical work. On a small scale, the gas production volume is minimal, and its rapid use and release eliminate any risk of explosion, ensuring operational safety. Several factors influence the exhaust gas volume and system efficiency, including the shape of the electrodes, the choice of electrolyte material, the type of fluids used, and the fluid temperature. For example, in the HEFR technique, increasing the temperature of the electrolytic solution enhances the rate of electrolysis by promoting ion movement and improving electrolyte conductivity. Similarly, elevated temperatures accelerate the rate of the chemical reaction. When combined, these processes boost gas production. It is important to note that this article focuses solely on introducing this new contraction method inspired by natural muscle contraction and does not delve into optimizing efficiency or maximizing gas output.

### 2.3. Harvest the Energy From HEFR Technique

The introduction discusses various methods of electricity generation, each with its own advantages and disadvantages. Among them, two methods—magnetic‐based and chemical‐based—are deemed superior and more applicable. The conversion of mechanical energy into electrical energy in this system is achieved through an electrochemical mechanism. In this process, two electrodes are embedded within a cell structure and are separated by an ion‐conducting membrane, while the electrolyte medium provides the necessary pathway for ion transport. As a result of ionic migration between the electrodes, an electric current is generated. It should be noted that, in the absence of mechanical motion, an initial voltage can still be produced; however, the corresponding current rapidly decays to zero due to concentration aspect and electrochemical equilibrium. To sustain a continuous current, mechanical agitation or movement of the electrolyte is required to maintain effective ion distribution. In other words, electrical energy can drive chemical reactions and induce significant mechanical effects through electrolysis, and conversely, mechanical motion can facilitate electrochemical reactions that lead to the generation of a stable electric current.

This research utilizes flexible aluminum and copper strips as anode and cathode electrodes, respectively. Liquid water, enhanced with salt and acetic acid, serves as the electrolyte. The presence of the electrolyte enables the measurement of voltage at the electrodes, which varies depending on the materials used for the electrodes and the electrolyte composition. Moreover, connecting multiple cells in series results in an increased voltage. A notable observation is that in a stationary solution, the electric current diminishes rapidly as a layer of ions coats the metal surfaces, obstructing ion movement. However, when the fluid is stirred or the electrodes are agitated, ion movement resumes, thereby increasing the electric current. Thus, the movement of ions is crucial for generating an electric current, necessitating an external stimulus to sustain it. In summary, by passing a dynamic fluid between the two electrodes, both voltage and current can be generated. This principle underpins the finalized version of the proposed mechanism.

### 2.4. Proposed Mechanisms

Usually, changes in concentration, pressure, and temperature in the fluid cause fluid movement. In the present study, the method of creating a pressure difference model has been used, which causes a change in volume and ultimately power transmission. Considering previous works and the introduction of the HEFR technique, an ideal method for creating a suitable pressure difference compared to the total weight of the mechanism, based on a combination of chemical reaction and electrolysis, this method has also been used here to create a pressure difference. In this research, instead of using wire and needle to create electrolysis, two very flexible strips of copper and aluminum were used. Although the thin metal strip can slow down the rate of electrolysis, it is very suitable for generating electricity from one side. The HEFR engine has been changed by replacing these two strips as electrodes for electrolysis and also for generating electricity when the system works in the opposite direction (moving the artificial muscle and generating electricity). The considered mechanism in this study is equipped with the HEFR technique to generate the energy necessary for contraction, which has been modified in the structure so that in addition to generating energy for contraction, there is also the possibility of generating electricity during muscle relaxation. Also, a mechanism has been adopted to imitate natural myosin so that contraction and relaxation are possible, and the movement of artificial myosin can be in the form of a curved bending. In general, the present mechanism includes the transfer of fluids A and B and their chemical reaction in a bag, which is also accompanied by an electrolysis reaction. Increasing pressure causes the bag to change shape, and mechanical work is achieved. The combination of two artificial myosins can cause reciprocating movement. The advantages of the proposed method compared to other methods include:

• Manufacturing cost: The production of soft actuators is considerably cheaper than that of electric motors. The raw materials required for soft actuators consist mainly of simple plastics and silicone‐based materials such as rubber and elastic tubes, whereas electric motors require metals, copper wire, carbon brushes, and other complex components.
• Weight of the soft actuator: Many advantages of this technology arise from its very low weight. Due to the necessity of using metal components, electric motors are significantly heavier than soft actuators, which are fabricated from lightweight and simple materials.
• Simplicity in construction: Due to the reduced number of mechanical parts in comparison with electric motors, greater simplicity in construction is achieved, which also results in a longer service life.
• Mimicking nature: The most important point that should not be overlooked is that nature provides far smarter and more efficient solutions; consequently, billions of living beings successfully employ mechanisms developed in nature. This research has also been inspired by natural systems and has utilized this inspiration in two forms: the development of an actuator with a mechanism close to natural muscle function and the use of reactive compounds and electrolysis to activate the artificial muscle.

Based on the mentioned advantages, three mechanisms have been considered for this study, which will be complementary in order. The first mechanism is a simple soft actuator, the second mechanism is a reciprocating muscle and the contraction action, and the third mechanism is the reciprocating movement of the artificial myosin in the form of a curved bending, which will be more close to natural myosin movement. In the following, each of the mechanisms, along with their introduction and experimental tests, will be presented.

## 3. Results

In this section, three mechanisms, based on the HEFR technique, are presented in which performance and harvesting of energy electricity are reported as follows.

### 3.1. Motion of HEFR Soft Actuator With Ability to Harvest Energy

**Figure 3:**
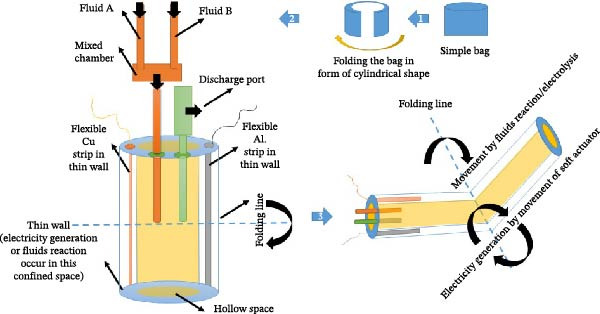
Schematic of experimental model of HEFR soft actuator with the ability to harvest energy.

Figure 3 presents a simple soft actuator capable of harvesting electricity. As illustrated in the figure, a rectangular nylon bag is folded into the shape of a thin‐walled hollow cylinder. Two primary fluid tubes and two thin metal strips are embedded within its walls. In the inlet line, two substances are mixed, leading to a rapid reaction between fluids A and B. This reaction results in a phase change and an increase in pressure within the combined fluid. During the mixing process, electrolysis is employed—not only to break fluid bonds but also to heat the fluid and accelerate the reaction. The pressure buildup causes the nylon bag, folded into a thin‐walled cylinder, to unfold into a straight, hollow cylindrical shape. This transformation in shape, driven by pressure changes and alterations in volume, enables the actuator to exert significant force. Additionally, the outlet line is designed to facilitate fluid ejection, allowing the bag to return to its relaxed state. Each line is controlled by a small solenoid, which can be opened or closed based on commands.

**Figure 4:**
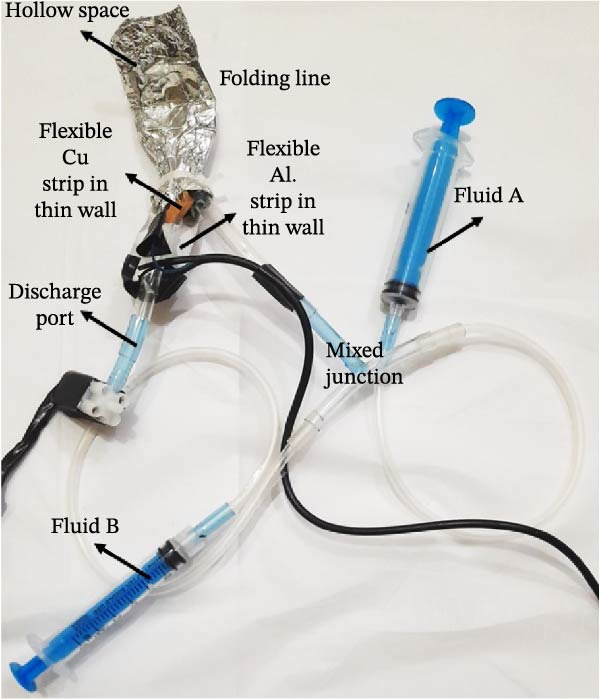
Experimental model of HEFR soft actuator.

**Figure 5:**
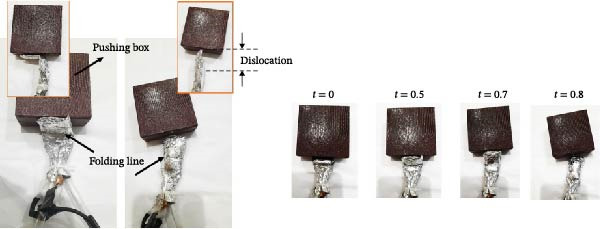
Performance of the HEFR soft actuator for pushing the weights.

**Figure 6:**
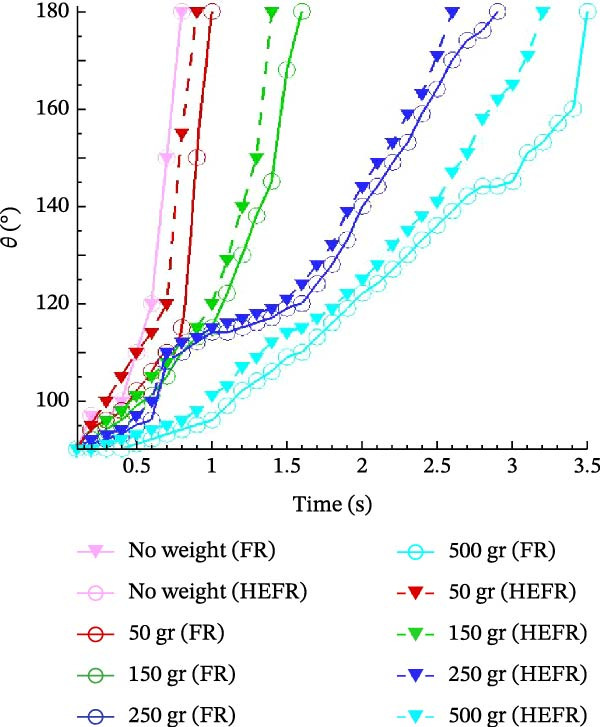
Variation of angle of soft actuator rotation to response time for different loads considering two experiments including HEFR technique and pure fluid reaction (FR).

In relaxation mode, or when no chemical reaction has occurred, any movement of the muscle caused by external forces such as wind or sea waves can generate electricity. This phenomenon opens the possibility of creating a new generation of electricity‐generating devices. The electricity is produced due to the interaction of two thin, flexible metal strips and an electrolyte solution, which generates a current in response to the muscle’s movement. As shown in Figure 4, the experimental model was constructed according to the specifications detailed in Table 2 and Figure 5. The model begins in a bent position at a 90° angle and is supported on a box designed to hold various weights. When the chemicals combine, the model exhibits rapid movement with a tendency to return to a horizontal position, pushing the box to a 180° angle. This reaction is driven by the interaction between two substances, leading to the release of energy. The test was conducted under two conditions: without electrolysis and with electrolysis. In the second condition, the electrolysis process generates gas, heats the solution, and increases the reaction rate, which accelerates the artificial muscle’s movement. Figure 6 illustrates the performance of this muscle in pushing different weights under both conditions: chemical reaction alone and the HEFR mode. The results highlight that in HEFR mode, and there is a slight reduction in response time. When the weight to be pushed increases, the response time lengthens, making the effect of electrolysis more significant. A nonlinear trend is observed in the muscle’s rotation rate from 90° to 180°. Initially, the speed increases gradually, but it then accelerates sharply, maintaining a faster rate. It is noteworthy that as the load increases, the response time also becomes longer. This is because the amount of chemical injection remains constant. For instance, pushing a 500‐g weight takes approximately three times longer than without the weight. The relationship between load and response time is also nonlinear. This study does not include a quantitative analysis of the parameters or an optimization process. Instead, the focus is on developing a mechanism that mimics natural systems.

**Table 2:** Characteristics of HEFR soft actuator and chemical materials.

Size of soft actuator (mm)	Volume of flow injection	Size of metal strips	Voltage of electrolysis	Electrolyte	Fluids A and B
60 × 30	1.5 cc (A) 0.5 cc (B)	10 × 60 (Cu) 20 × 60 (Al)	12 v 1 (amp)	A, B, NaCl	Acetic acid (A: CH_3_COOH), bicarbonate (B: NaHCO_3_), water (H_2_O)

**Figure 7:**
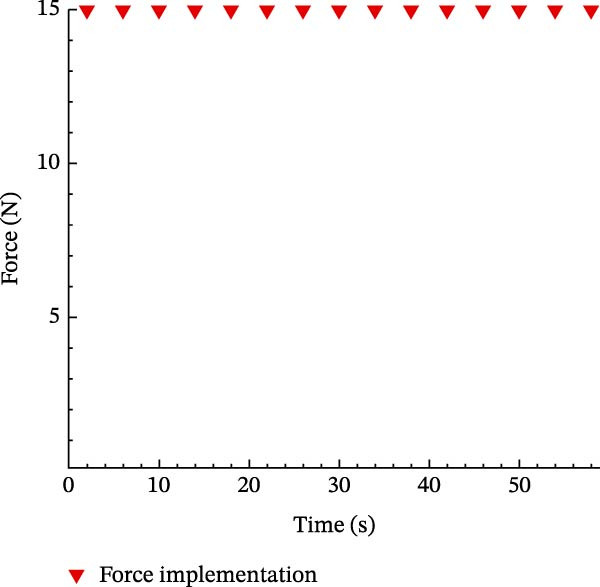
Implementation of external force on HEFR soft actuator for harvesting the energy.

**Figure 8:**
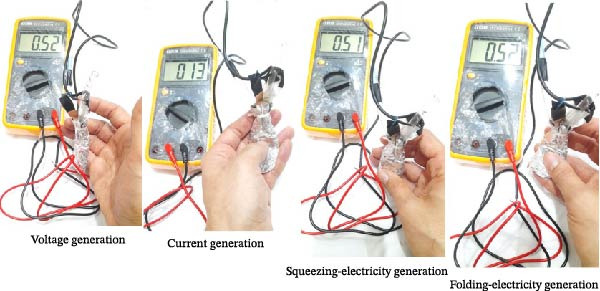
Generating of voltage and current from the HEFR soft actuator.

**Figure 9:**
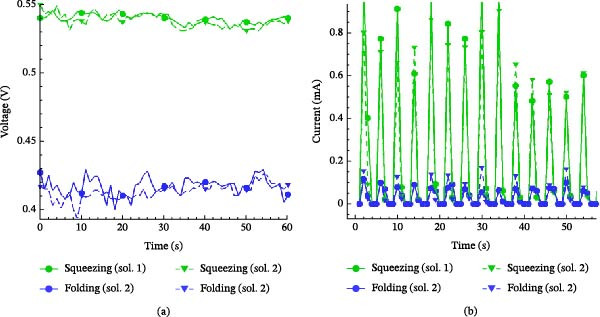
Variation of voltage (a) and current (b) generation to respond time for two cases of squeezing and folding the bag considering two weak and strong solutions.

One potential achievement during muscle relaxation is the generation of electricity through the movement of a soft actuator. This principle is akin to an electric motor, which produces mechanical work when consuming electricity. Similarly, when the electric motor’s shaft rotates, electricity can be generated as an output. Applying this concept, the movement of a muscle can generate electricity based on the principle of an electric cell. Using two electrodes, made of aluminum and copper, along with a suitable electrolyte, it becomes possible to produce electricity and achieve a suitable voltage. However, it is noteworthy that due to the accumulation of ions on the electrode surfaces, the electric current diminishes rapidly to zero. To prevent this, either fluid movement or electrode motion is necessary. In this study, four experiments were conducted to explore this method. In the first experiment, the muscle was compressed with a force of 15 g, while in the second experiment, the muscle was folded using the same force. These actions were repeated in an oscillatory manner for two different electrolytes, whose specifications are provided in Table 1 (refer to Figures 7 and 8). The voltage produced and the resulting current from these operations were measured. The results, shown in Figure 9, indicate that the voltage generated by the first electrolyte solution was higher, attributable to its greater concentration. The experiments reveal that solution concentration significantly impacts electricity production through this method. Furthermore, the type of motion—whether squeezing or folding—does not notably affect the results; only the movement of fluid is required for electricity generation. These findings suggest that muscle movement can indeed produce electricity. Although the current production rate is ~5.5 mW, optimization of parameters, adjustments to geometry, and the use of series or parallel connections for each muscle could potentially increase this output. This study aims to present a mechanism inspired by nature for electricity generation, though optimization has not been the focus of this research.

### 3.2. Sliding Contraction of Artificial Filament Mechanism

**Figure 10:**
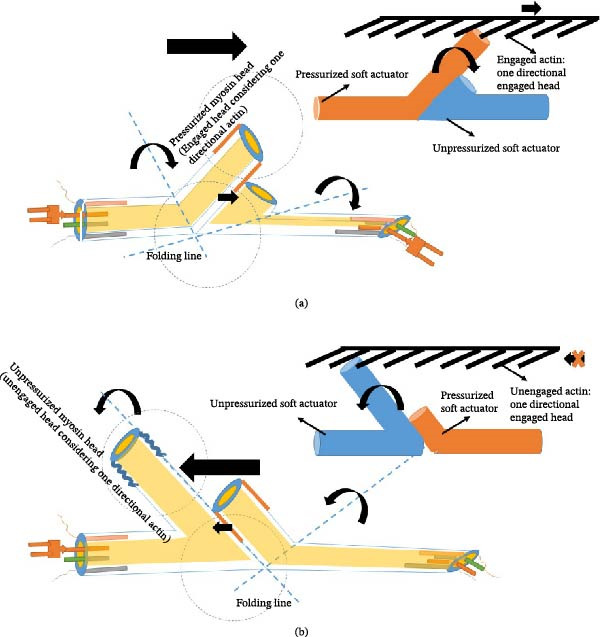
Schematic of experimental model of reciprocating HEFR soft actuators with the ability to harvest energy for contraction (a) and relaxation (b) modes.

One issue with the above mechanism is the absence of a defined return path for the artificial myosin. In other words, during the powerful forward movement, the return path of the myosin and the repetition of this motion must be observed. In nature, this process seems to rely on the properties of elastic materials. More physiological research is needed to explore this further. In practical terms, creating such a system involves employing another muscle, as in previous models, to generate a reciprocating motion. It is crucial to note that if the reciprocating movement is completely symmetrical, no net movement will occur. This is because the forward motion will be counterbalanced by an equal backward motion, resulting in zero overall displacement. To address this challenge, the principle of asymmetric reciprocating motion is utilized. This is achieved by altering the folding locations of the two artificial muscles, as illustrated in Figure 10. The first artificial myosin is folded in half, with the hinge positioned at the midpoint of the structure, while the second artificial myosin is folded at the first quarter of its length. Consequently, although both perform movements within the 0–90° range, the first myosin head lacks support in its relaxed state. Due to its softness, it easily slides over the actin without engaging. However, during movement, the increased pressure and stiffness of the artificial myosin head cause it to engage with the actin, resulting in motion. To enhance engagement, the actin is designed as one engaged directional strip. This configuration allows myosin to move smoothly on one side while engaging with the edges of the strips on the other side. As a result, the back‐and‐forth motion between the artificial actin and myosin becomes asymmetrical. The combination of these two factors—the asymmetry in the reciprocating motion of the artificial myosin and actin—ultimately generates contraction.

**Figure 11:**
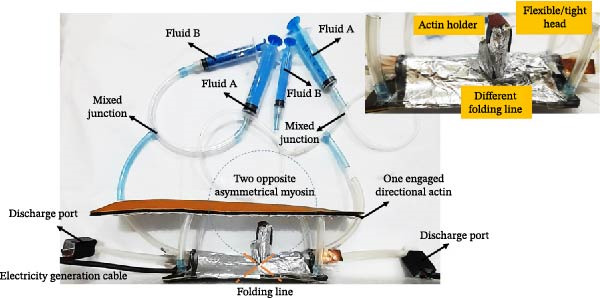
Experimental model of reciprocating HEFR soft actuators.

**Figure 12:**
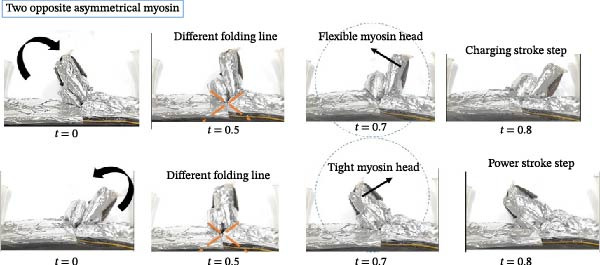
Performance of reciprocating soft actuators considering the response time, in which the mechanism is inspired from natural myosin. The marked lines in this mechanism are in the different folding lines, in which the mechanism provides a variable artificial myosin head: flexible head (in charge action) and tight head (in pressure stroke action).

According to the schematic presented, the experimental model of the described concept has been constructed based on Table 3 and Figure 11. In this model, two artificial myosins are positioned opposite each other, designed with distinct folding lines. These folding lines enable one head to function flexibly and the other to behave rigidly, adhering to asymmetrical principle 1. Furthermore, the actin, oriented in one direction, slides smoothly across the myosin during its movement and engages effectively in the opposite direction, facilitating contractile motion as defined by asymmetrical principle 2. Chemical injection is performed using a syringe, while the opening and closing of outlet valves are regulated by solenoid valves. Once the chemical fluids are mixed, the outlet valve is closed to create increased pressure. Figure 12 illustrates the reciprocating motion of the opposing artificial myosins. As shown, the movement from an angle of ~90°–180° is depicted for both myosins, with each stroke taking around 1 s. Notably, the myosin head, which is folded halfway, demonstrates variability: It operates as a flexible head during the charging step and transitions to a rigid head during the power stroke.

**Table 3:** Characteristics of sliding contraction of artificial filament mechanism.

Size of soft actuator (mm)	Size of actin (mm)	Volume of flow injection (cc)	Size of metal strips (mm)	Voltage of electrolysis
60 × 30	200 × 45	1.5 (A) 0.5 (B)	10 × 60 (Cu) 20 × 60 (Al)	12 (v) 1 (A)
Fluids A and B	Electrolyte	Lines of folding (mm)	Displacement for each actuation (mm)	
Acetic acid (A: CH_3_COOH), bicarbonate (B: NaHCO_3_), water (H_2_O)	A, B, NaCl	30 and 15	15	—

**Figure 13:**
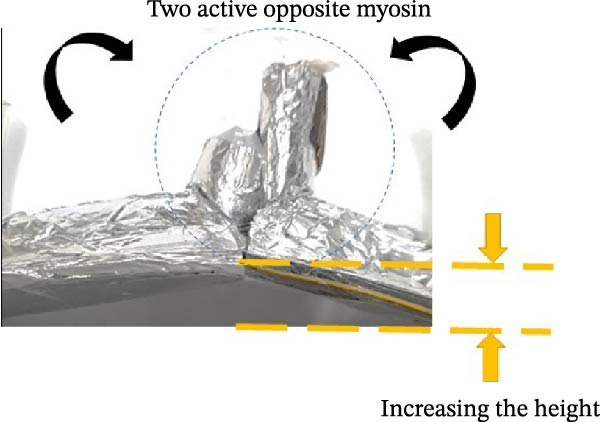
Increasing the height of myosin filament in the case of the function of both opposite artificial myosins simultaneously and finding the actin.

During the movement of M1‐myosin (folded at half its length) in the power stroke, the artificial myosin head demonstrates stiffness and efficient power transmission. This phase enables the head to pull on actin, resulting in contraction. Conversely, when M1‐myosin is discharged by releasing gases through the discharge port, the M2‐myosin (folded at a quarter of its length) is activated by chemical entry and FRs as its exit valve closes. The increased pressure causes M2‐myosin to expand, compressing M1‐myosin and driving its rotation through an angle of ~90°–180°. Importantly, this movement shifts the body of M1‐myosin, while its head remains flexible and pressure‐free, allowing it to pass through actin without interference. This asymmetry, termed the asymmetric principle, is a critical concept discussed in detail within this article. Additionally, by enabling variable myosin head positioning through alternating actions of the two myosins, simultaneous operation achieves greater regulation and facilitates actin engagement. This dual functionality increases the height of the two myosins, providing an effective method for actin interaction. While the precise mechanism by which the myosin head locates actin and transfers force efficiently in nature remains unclear, the present method offers a practical solution for actin targeting. As illustrated in Figure 13, the simultaneous operation of both myosins results in a height increase of ~30 mm for the myosin head.

**Figure 14:**
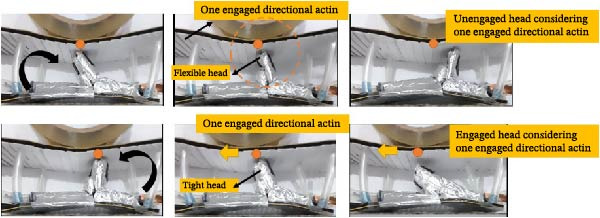
Reciprocating function of two opposite soft actuators with different folding lines considering one engaged directional actin head. Unengaged stage: passing the myosin from actin due to the flexibility of the head (unpressurized head) and unengaged head of actin. Engaged stage: due to stiffness of myosin head (pressurized head) and one engaged directional actin head, the engagement has been formed, and the displacement of actin and weight results in almost 15 mm.

**Figure 15:**
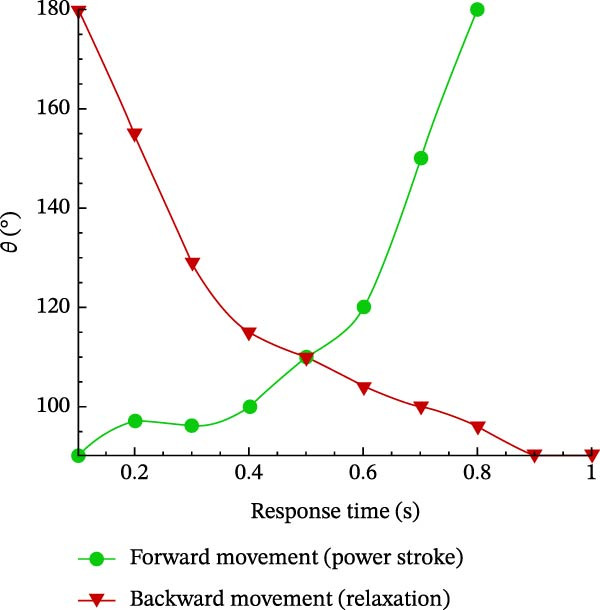
Contraction/relaxation of actin to response time.

As shown in Figure 14, the rotation rate of the artificial myosin over time in two reciprocating modes is ~90° per second. The proper performance of this mechanism results from the transition from the charging step to the power stroke, ultimately producing contraction. In the first movement, the M2‐myosin moves the primary myosin (M1‐myosin) backward. Due to the flexibility of the main myosin head, it easily passes through the actin without causing any significant contraction. During the subsequent movement, pressure in the main myosin increases, tightening the myosin head, which then moves at an angle of 90°–180°. This motion, influenced by the tightening of the myosin head (asymmetric principle 1) and the edged configuration of the actin filaments (asymmetric principle 2), results in engagement and the formation of a proper connection. This interaction causes the actin to move, leading to effective contraction. The movement of the artificial myosin not only facilitates the displacement of the actin but also supports a weight of up to 200 g. Although the displacement in a single cycle is ~15 mm, repeated actions leverage the asymmetric principles to achieve a desirable contraction, akin to that of natural muscles. Figure 15 illustrates the relationship between the rotation of myosins and response time. It clearly demonstrates that the contraction behavior of the soft actuator, when moving forward and backward, is entirely nonlinear.

**Figure 16:**
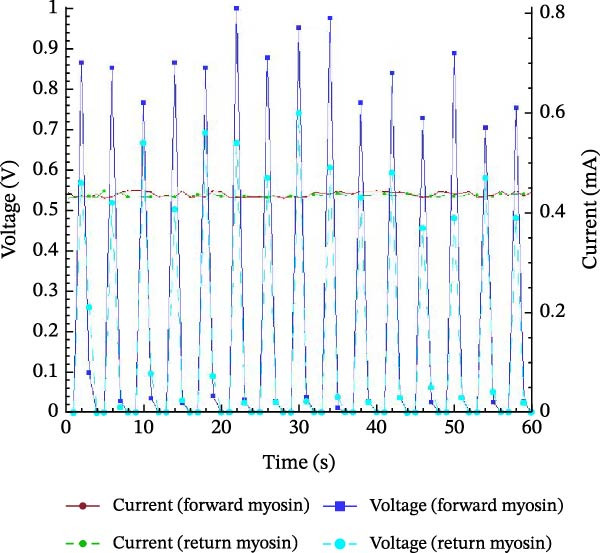
Voltage (left column) and current (right column) generation under folding the myosins.

When the muscle is in a relaxed state, without any chemical reactions or electrolysis, a small amount of electricity can be produced by moving the muscle laterally. Figure 16 illustrates the current and voltage generated by a reciprocating muscle. Although the power output is minimal, the concept of utilizing multiple muscles to increase electricity generation is practical. Similar to the observations in previous sections, any movement of an artificial muscle can generate electricity, while the muscle is in its relaxed state.

### 3.3. Multicontractile Module and Camber Movement of Artificial Myosin

**Figure 17:**
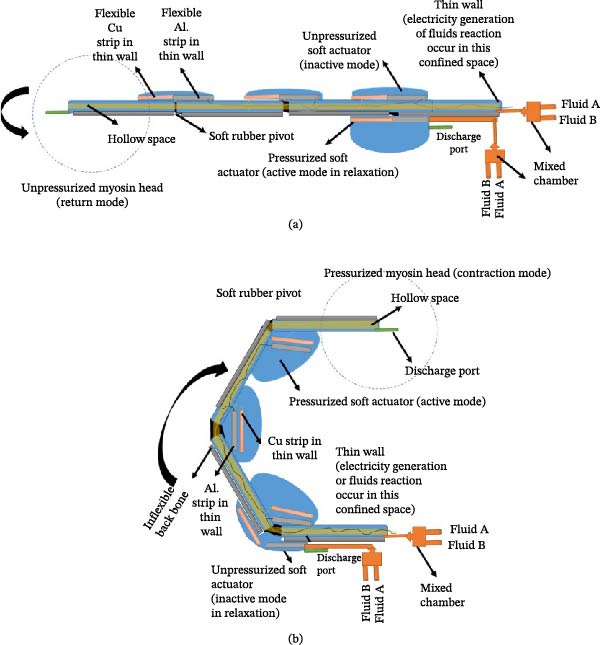
Schematic of experimental model of multicontractile module of HEFR soft actuators with the ability to harvest energy for relaxation (a) and contraction states (b).

**Figure 18:**
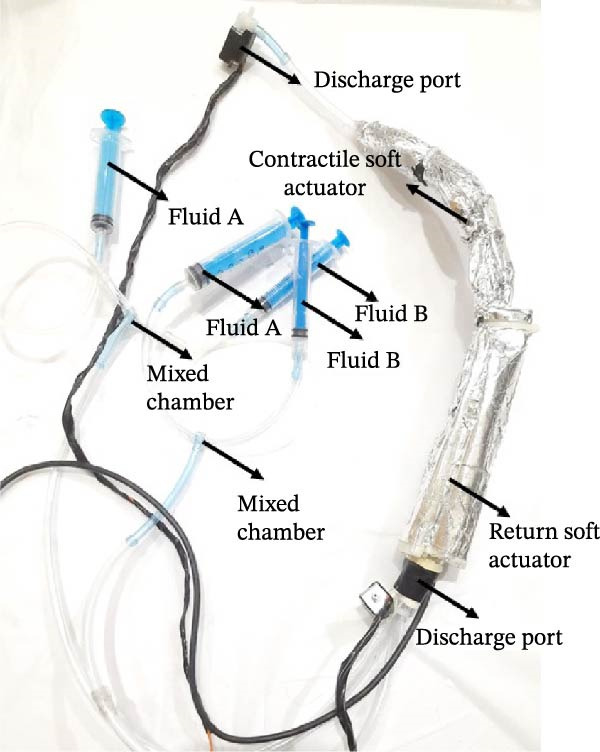
Experimental model of the multicontractile module of the HEFR soft actuator.

**Figure 19:**
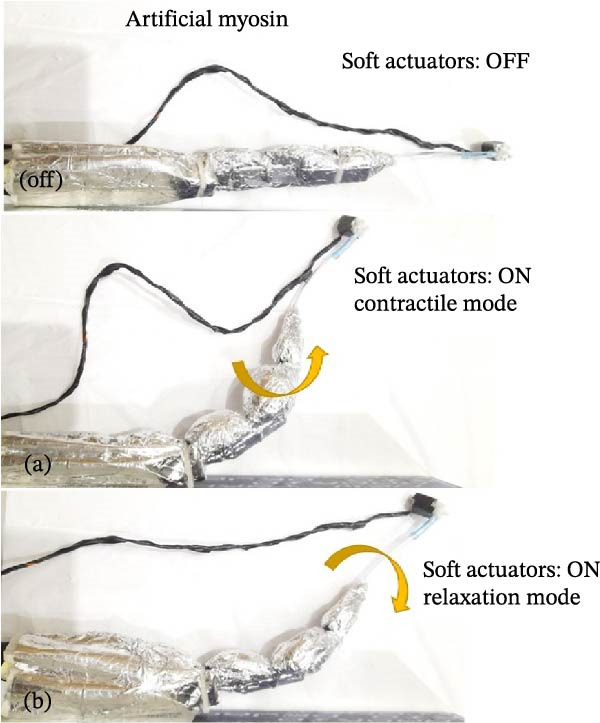
Performance of artificial myosin considering multiple soft actuators in contraction (a) and relaxation mode (b).

**Figure 20:**
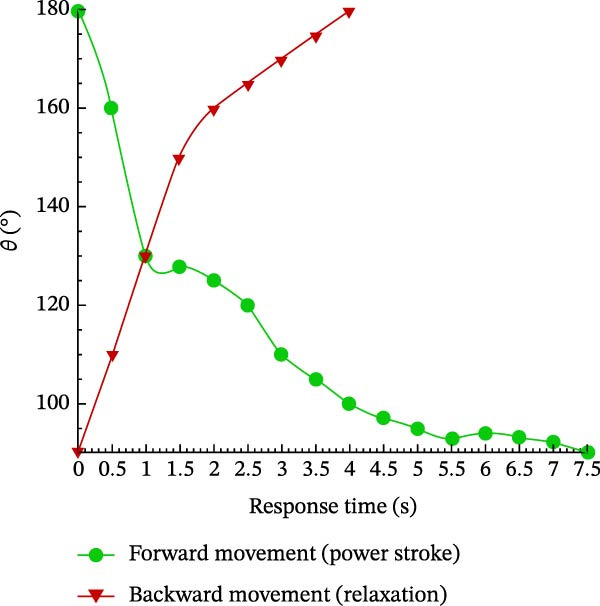
Contraction (power stroke)/relaxation of multiple soft actuators to response time.

**Figure 21:**
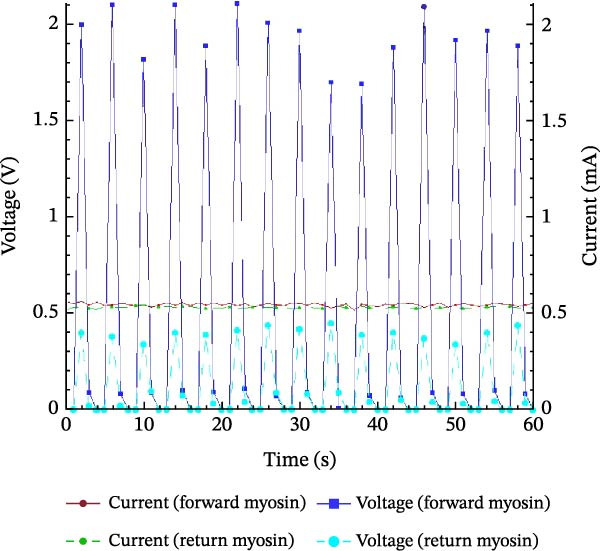
Voltage (left column) and current (right column) generation under folding the myosins for both multiple soft actuators and return soft actuator.

Another important point is that the movement of myosin does not resemble that of a lever. In practice, when myosin moves towards actin, it adopts a curved and flexible form. Specifically, the myosin stalk bends at multiple points, which is characteristic of natural myosin behavior. Inspired by this observation, instead of incorporating a single fold (soft hinge) in artificial myosin, multiple folds should be applied, as illustrated in Figure 17. To achieve bending under increasing pressure, the stiffness at the hinge locations must differ from that of the surrounding structure. This asymmetry creates a tendency to bend towards the side with lower stiffness, leading to contraction at each fold location, ultimately giving the artificial myosin a curved shape. Furthermore, an artificial muscle is positioned along the return path, with uniform stiffness in all directions. This design ensures the muscle remains horizontal rather than adopting a bent shape. The artificial myosin head functions similarly to natural myosin, remaining soft in its relaxed state and becoming stiff during contraction under increased pressure. The mechanism for the artificial myosin closely mirrors the behavior of real myosin, utilizing a chemical/electrolysis reaction for bending and asymmetrical movement. Based on these principles, an experimental model has been developed (Figure 18 and Table 4) that can bend to an angle of ~90° or more and return to a horizontal position. The model employs three soft actuators for the bending motion and one soft actuator for horizontal stabilization. As shown in Figure 19, the artificial myosin transitions from a horizontal form to a bent shape by activating the three soft actuators, which account for differing stiffness in the waist and abdomen sections (Figure 19a, asymmetrical principle 3). Activating the return soft actuator allows the artificial myosin to revert to its original horizontal position (Figure 19b). Figure 20 illustrates the rotation of the artificial myosin over time. The graph indicates that, due to the consistent volume of chemical reaction fluids and prior testing scenarios, the response time increases as the forward soft actuators’ volume size is enhanced. In addition, Figure 21 plots the voltage and current produced during the folding of the artificial muscle, considering both the upper and lower soft actuators.

**Table 4:** Characteristics of multicontractile module.

Size of each soft actuator (mm)	Number of soft actuators	Volume of flow injection (cc)	Size of metal strips (mm)	Voltage of electrolysis	Electrolyte	Fluids A and B
60 × 30	3 (forward M.) parallel 1 (return M.)	1.5 (A) 0.5 (B)	10 × 60 (Cu) 20 × 60 (Al)	12 (v) 2.5 (A)	A, B, NaCl	Acetic acid (A: CH_3_COOH), bicarbonate (B: NaHCO_3_), water (H_2_O)

Based on the mentioned results and explanations, the suggested mechanism, according to the natural contractile mechanism, would be several applications in various fields, such as flapping wings by consuming electric current and chemical reaction or, vice versa, generating electricity from the movement of wings powered by wind or ocean waves.

## 4. Conclusion

This article investigates the performance of a soft actuator designed to mimic artificial myosin, using the HEFR method. Based on the quantitative and qualitative results, it demonstrates that the HEFR method is capable of muscle movement or generating energy from muscle motion, making it a viable candidate for artificial muscle applications. Furthermore, this method is scalable, enabling the production of actuators in various sizes. Three models were proposed and examined:

1. First Model: A simple smooth muscle was analyzed. It was observed that as fluid pressure increased, the muscle stretched, significantly reducing the time required to lift a weight. This review highlights the practicality of combining electrolysis and chemical reactions, particularly as the weight increases.
2. Second Model: The limitations of the first design were addressed by using two opposing myosins working reciprocally. This design incorporated two asymmetric principles to achieve both flexibility and rigidity in the myosin head during power charging and transfer. An actin strip with one‐directional edges was introduced, mimicking natural myosin contraction.
3. Third Model: To replicate the curved motion observed in natural myosin, a multisoft actuator was utilized. The stiffness of the waist and abdomen regions varied, resulting in a forward motion similar to natural myosin during both the power stroke and relaxation phases.

In all cases, the possibility of harvesting electricity was demonstrated. This approach not only enables displacement but also facilitates electricity generation during the displacement process. While the proposed method closely emulates natural mechanisms, it holds significant potential for optimization and development across various applications.

## Funding

No funding was received to assist with the preparation of this manuscript.

## Conflicts of Interest

The authors declare no conflicts of interest.

## Data Availability

All of the data will be available upon reasonable request.
